# Targeted Therapies Combined with Intensive Chemotherapy in Fit Acute Myeloid Leukemia: Past Developments, Current Evidence, and Future Therapeutic Paradigms

**DOI:** 10.3390/jcm15124529

**Published:** 2026-06-11

**Authors:** Matteo Molica, Laura De Fazio, Claudia Simio, Caterina Alati, Massimo Martino, Marco Rossi

**Affiliations:** 1 Department of Hematology-Oncology, Azienda Ospedaliera Renato Dulbecco, 88100 Catanzaro, Italy; defalaura@gmail.com (L.D.F.); rossim@unicz.it (M.R.); 2 Hematology and Stem Cell Transplantation and Cellular Rerapies Unit (CTMO), Department of Hemato-Oncology and Radiotherapy, Grande Ospedale Metropolitano “Bianchi-Melacrino-Morelli”, Presidio Morelli, 89121 Reggio Calabria, Italy; caterina.alati@gmail.com (C.A.);

**Keywords:** acute myeloid leukemia, target therapies, fit patients

## Abstract

Acute myeloid leukemia (AML) is a genetically and clinically heterogeneous hematologic malignancy in which intensive induction chemotherapy remains the standard therapeutic platform for medically fit adults. In recent years, however, the frontline treatment paradigm has progressively evolved from a purely cytotoxic approach toward a biologically informed strategy. This shift has been driven by the identification of recurrent molecular alterations—particularly FLT3 and IDH1/2 mutations—as well as renewed interest in antibody-based therapies and the growing recognition that relapse, resistance, and measurable residual disease (MRD) are shaped by clonal architecture rather than blast burden alone. This review examines the development of targeted therapies combined with intensive chemotherapy in AML. We discuss the biological rationale for combination approaches and summarize the key clinical studies that have defined current practice, including trials evaluating FLT3 inhibitors, gemtuzumab ozogamicin, IDH inhibitors, and venetoclax-based strategies. We also address the role of targeted therapy across different treatment phases, including induction, consolidation, and post-remission settings, and analyze emerging data regarding MRD-guided treatment strategies, mechanisms of resistance, and integration with allogeneic hematopoietic stem cell transplantation. The integration of targeted agents with intensive chemotherapy is reshaping frontline AML therapy and represents a critical step toward precision medicine. While genotype-directed strategies—such as FLT3 inhibition—have already demonstrated survival benefit, optimal patient selection, treatment sequencing, and duration remain areas of active investigation. Future progress will likely depend on MRD-driven treatment adaptation, improved understanding of clonal evolution, and the development of rational multi-agent combinations capable of achieving deeper and more durable remissions.

## 1. Introduction

AML remains one of the most challenging adult leukemia because initial chemosensitivity and long-term disease control are often dissociated. Standard anthracycline-cytarabine induction can induce morphologic complete remission in a large proportion of younger and medically fit adults, yet many of these patients later relapse because chemotherapy does not uniformly eradicate leukemic stem and progenitor compartments [1]. Historically, therapeutic progress in fit AML was pursued by intensifying existing drugs, optimizing the daunorubicin dose, refining cytarabine schedules, and improving transplantation algorithms [1]. These steps mattered, but they did not solve the core biologic problem: relapse in AML reflects both the persistence of molecularly defined leukemic subclones and the dynamic process of clonal evolution, whereby therapeutic pressure selects or reshapes resistant populations over time.

The genomic era changed this framework. Sequencing studies showed that AML is assembled from cooperating lesions in signaling genes, transcription factors, chromatin modifiers, cohesin components, tumor suppressors, and metabolic enzymes [2,3]. This complexity immediately suggested both opportunity and caution. The opportunity was obvious: if some AML subgroups are driven by targetable molecular dependencies, adding a targeted agent to chemotherapy might deepen responses, reduce minimal residual disease, and delay or prevent relapse [2,3]. The caution was equally important: a mutation can be recurrent without being uniformly truncal, a kinase can be biologically central without being pharmacologically suppressible in vivo, and a targeted drug can have single-agent activity without necessarily improving survival when layered onto intensive treatment [2,3].

The case for combination rather than replacement was strong for several reasons. First, cytotoxic chemotherapy still produces rapid debulking and remains the most reliable means of achieving prompt marrow clearance in proliferative disease [1,4]. Second, many targeted agents are most effective when residual disease burden is already reduced. Third, AML resistance mechanisms frequently involve clonal adaptation rather than simple target persistence; combining chemotherapy with targeted inhibition may therefore narrow the evolutionary space available to surviving clones [2,3]. Fourth, the post-remission setting offers a biologically distinct opportunity: an agent that is only modestly active in overt relapse may still be highly valuable when used to suppress low-level residual disease after induction or transplantation.

Yet, combining targeted agents with intensive therapy is intrinsically complicated. Questions of sequencing, dose intensity, overlapping myelosuppression, hepatotoxicity, cardiac safety, antifungal interactions, and transplant timing all become relevant [4]. Moreover, frontline trials must account for the fact that fit AML patients often proceed through multiple treatment phases—induction, re-induction when needed, consolidation, transplant, and sometimes maintenance. A targeted strategy may fail if judged only at the induction stage, even though its principal benefit lies in post-remission disease control. Conversely, a higher remission rate does not necessarily translate into longer survival if toxicity, treatment delays, or resistant relapse negate early gains.

For these reasons, the most useful way to view targeted therapy with intensive chemotherapy is not as a collection of isolated drug additions, but as the gradual re-engineering of the entire frontline AML pathway. In some subgroups, such as FLT3-mutated disease, that re-engineering has already altered standard practice. By contrast, IDH inhibitor-based and venetoclax-based intensive combinations remain promising but less mature, with current limitations including relatively small and heterogeneous datasets, limited randomized evidence, uncertainty regarding optimal sequencing and patient selection, and insufficient long-term data on response durability, MRD clearance, toxicity, and post-remission management [5,6]. Across all subgroups, measurable residual disease (MRD) assessment, increasingly sensitive genomic monitoring, and a more nuanced understanding of clonal fitness are changing the questions we ask in clinical trials [7,8]. Accordingly, future treatment strategies should aim not only to induce morphologic remission, but also to achieve deeper disease clearance, as reflected by MRD negativity and, when applicable, molecular remission, while preserving tolerability and enabling effective post-remission interventions.

This review focuses on fit adults receiving standard chemotherapy as the therapeutic backbone. It does not attempt to summarize the entire targeted therapy literature in older or unfit AML, although that experience-especially with venetoclax and IDH inhibitors-has materially influenced thinking in the fit setting [5,6]. Instead, the emphasis is on how targeted therapies have been combined with induction and consolidation chemotherapy, what has worked, what has failed, and which future strategies are most likely to define the next phase of frontline AML therapy [9,10].

## 2. From Empiricism to Biologic Targeting

The earliest era of targeted therapy in AML was defined less by precision than by aspiration. Investigators recognized that cytotoxic intensification alone was unlikely to meaningfully change long-term outcomes and began to explore whether antibodies, differentiating agents, or signal transduction inhibitors could increase the depth of remission [9,11,12]. Gemtuzumab ozogamicin became the first major success and the first major warning. Targeting CD33 made biologic sense because the antigen is expressed on most AML blasts, and the antibody–drug conjugate format offered a way to deliver potent cytotoxic payload directly to leukemic cells [13,14]. Early clinical enthusiasm led to regulatory approval, but toxicity concerns—particularly hepatic sinusoidal injury—clouded its frontline development [13]. Retrospectively, the gemtuzumab story taught several enduring lessons. Target choice matters, but schedule and dose matter just as much. Frontline combinations are not rescued by good biology if toxicity prevents delivery of subsequent therapy. In addition, subgroup benefits can be substantial even when global trial results appear mixed [14,15].

The re-emergence of gemtuzumab with fractionated dosing and more careful patient selection was therefore conceptually important [14,15]. It showed that a targeted agent could be rehabilitated through better pharmacologic design rather than abandoned as a failed principle. It also highlighted an issue that still recurs in AML: the effect of a targeted therapy is often distributed unevenly across biologic subsets. Patients with favorable and some intermediate-risk features seemed to benefit most, whereas adverse-risk disease often remained dominated by underlying resistance biology [14,15]. This pattern foreshadowed the later experience with mutation-directed therapy, where context-co-mutations, disease kinetics, stemness signatures, and transplant use modulate the value of the target itself [2,3,9].

In parallel, kinase inhibition entered AML with enormous promise but limited early success. FLT3 was an obvious candidate because FLT3-ITD mutations are common and strongly associated with relapse [16,17]. Yet first-generation FLT3 inhibitors struggled. Some compounds did not sustain plasma concentrations sufficient for continuous inhibition; others were too nonselective, too toxic, or too vulnerable to protein binding and chemotherapy-induced pharmacokinetic shifts [17,18]. Several early randomized studies were therefore disappointing [18]. These trials are sometimes remembered simply as negative, but that interpretation is too crude. They established that biologic target validation is not equivalent to therapeutic target tractability. They also clarified that mutation-defined frontline studies require highly standardized supportive care, careful timing of inhibitor exposure, and often correlative assays proving on-target suppression [17,18]. Without that, negative results are difficult to interpret.

A second historical theme was the mismatch between relapse activity and frontline utility. Sorafenib, for example, generated significant interest because of its anti-FLT3 activity and measurable clinical responses in some settings [18]. Yet its role was context-dependent, with benefits appearing more convincing in some younger cohorts and post-transplant maintenance settings than in others [18]. This again taught the field that targeted therapy in AML cannot be reduced to a single yes-or-no judgment. The same drug may have different values in overt leukemia, molecular residual disease, or maintenance after transplantation.

The pre-midostaurin period therefore produced a body of experience that was more constructive than is sometimes acknowledged [9,12,17,18]. It clarified the need for molecular selection, consistent target inhibition, and trial designs that follow patients across the entire treatment continuum. It also set expectations for what success should mean. A targeted agent added to chemotherapy should not merely improve blast clearance at day 14 or transiently increase remission rates; it should alter relapse biology and, ideally, survival. Those standards were eventually met in FLT3-mutated AML, but only after the field passed through a decade of instructive partial failures [19].

This historical arc also explains why the fit AML setting evolved differently from the unfit setting. In older or frailer patients, the therapeutic threshold for adopting a targeted or low-intensity combination is lower because standard intensive chemotherapy may not be feasible [9,20,21]. In fit AML, by contrast, any new regimen must outperform or at least meaningfully complement an already active chemotherapy backbone [1,4,6,19]. That is a higher evidentiary bar and one reason why progress in the fit population has seemed slower. It is also why each genuine success—gemtuzumab in selected groups, midostaurin in FLT3-mutated disease, and now newer combination platforms—has had outsized conceptual importance [14,15,19,22].

## 3. FLT3 Inhibitors: Proof of Principle and Beyond

Among all targeted strategies combined with intensive chemotherapy, FLT3 inhibition provides the clearest proof of principle. This is partly because FLT3-mutated AML is common in clinically high risk, but also because FLT3 biology offers a relatively direct therapeutic hypothesis [16,17]. Constitutive FLT3 signaling activates proliferative and antiapoptotic pathways, contributes to high leukemic burden, and is associated with a strong propensity for relapse [16,17]. In principle, intensive chemotherapy can debulk FLT3-mutated AML effectively, but unless signaling is durably suppressed, residual clones may rapidly re-expand. A successful frontline FLT3 strategy therefore needs to do more than produce remission: it must change the post-remission trajectory.

Midostaurin achieved exactly that [19]. Its importance lies not only in regulatory approval, but in what its trial established methodologically. The benefit of adding midostaurin was not reducible to a dramatic single time-point cytoreductive effect. Rather, the study showed that integrating a targeted agent throughout induction, consolidation, and continuation phases could translate into a survival signal in a molecularly defined population [19]. This had several implications. First, genotype should shape frontline therapy, not only salvage therapy. Second, intensive chemotherapy and targeted inhibition can be complementary rather than competitive. Third, survival benefit can emerge from cumulative effects across treatment phases, even when remission rates alone do not fully capture the magnitude of the benefit [9,19,23].

The success of midostaurin also sharpened interest in the biologic heterogeneity within FLT3-mutated AML. The allelic ratio, co-occurring NPM1 mutation, co-mutation burden, and transplantation strategy all influence prognosis [16,24,25]. Although contemporary risk frameworks have evolved, clinicians still confront practical questions: which patients derive sufficient benefit from chemotherapy plus FLT3 inhibition alone, which should proceed to transplant in first remission, and whether post-transplant maintenance should be routine [25,26]. In practice, the availability of a frontline FLT3 inhibitor has not simplified care so much as redefined the baseline from which these decisions are made. A fit patient with FLT3-mutated AML is no longer treated with generic 7+3 followed by generalized post-remission planning; instead, FLT3-directed therapy is embedded from the outset [19,22,25].

Second-generation FLT3 inhibitors have pushed this paradigm further. Quizartinib, with stronger selectivity for FLT3-ITD, reinforced the notion that sustained and potent FLT3 inhibition matters [22]. Its frontline trial strengthened confidence that the midostaurin result was not idiosyncratic [22]. At the same time, it raised new issues. The more potent and selective the inhibitor, the greater the need to think carefully about escape mechanisms, including kinase domain mutations, activation of parallel signaling pathways, and clonal shifts into non-FLT3-dominant disease states [22,27,28,29]. Potency is beneficial, but selective pressure can reshape relapse biology in ways that eventually require sequential or combinatorial strategies [27,28,29,30].

Gilteritinib, already important in relapsed FLT3-mutated AML, has intensified interest in whether the distinction between frontline and salvage FLT3 inhibition should blur [27]. If a potent inhibitor is clearly superior in relapse, should it be moved earlier? The answer is probably yes for at least some settings, but frontline adoption requires more than extrapolation from salvage efficacy. Intensive chemotherapy changes marrow kinetics, toxicity profiles, antifungal exposure, and transplant timing. A successful frontline regimen must therefore be evaluated as a full platform, not as a simple transfer of an active relapse drug into induction [19,27].

Another unresolved issue is duration. FLT3-mutated AML may require suppression across multiple disease states: at diagnosis, after remission induction, during consolidation, and possibly after transplant [19,22,26,29]. The traditional oncology logic of fixed treatment cycles sits uneasily with the biology of molecular residual disease [7,8,31]. This has led to a broader reconceptualization of targeted therapy in AML. For FLT3-mutated disease, the targeted agent may be less like a one-time adjunct and more like a disease-state-specific control mechanism deployed across the treatment continuum.

Combination strategies are the next frontier. FLT3 inhibition may synergize with venetoclax because FLT3-driven leukemias can be highly apoptosis-primed and because kinase inhibition may lower the threshold for mitochondrial cell death [6,32,33,34]. Triplet concepts that combine intensive chemotherapy, FLT3 inhibition, and venetoclax are therefore attractive, especially in biologically aggressive disease where two-drug platforms may still permit residual resistant clones [6,34,35]. The challenge is whether such regimens can be delivered safely and without compromising the ability to proceed to transplant. Here again, the fit AML setting imposes a high bar: deeper remission is valuable only if it does not cause excessive infectious morbidity or prolonged treatment interruption [6,34,35].

In summary, FLT3 inhibitors have already changed standard frontline therapy and remain the most mature model for how targeted therapy can be meaningfully integrated with intensive chemotherapy [19,22,25,29]. Their story demonstrates that molecular selection, sustained target inhibition, and longitudinal use across treatment phases can produce real survival benefit. It also illustrates the next problem AML must solve: once a target is successfully incorporated, how should that platform be optimized through maintenance, sequential inhibition, and biologically rational triplet combinations?

## 4. Gemtuzumab Ozogamicin and Antibody-Based Targeting

Although mutation-directed therapy now dominates the discussion of precision AML, antibody-based targeting deserves equal historical attention because it was the first strategy to show that biologically selected augmentation of chemotherapy could reduce relapse in fit patients [13,14]. Gemtuzumab ozogamicin remains uniquely instructive because its trajectory captured many of the conceptual and practical challenges that later confronted small-molecule targeted therapy. It targeted a broadly expressed leukemia antigen rather than a private mutation, linked selectivity to payload delivery rather than pathway inhibition, and required careful balancing of efficacy against organ-specific toxicity.

The early gemtuzumab experience initially seemed disappointing because toxicities overshadowed benefit [13]. However, later analyses and revised schedules showed that this was not a failure of the target itself but of implementation [14,15]. Fractionated dosing improved the therapeutic index and revealed that the drug could meaningfully reduce relapse in selected patients when added to standard induction and consolidation [14,15]. The lesson was profound: in AML, the success of a targeted adjunct may depend less on the conceptual elegance of the target than on whether the targeted therapy can be integrated without disrupting the rest of the treatment pathway [9,14,15].

Another reason gemtuzumab matters is that it broadened the idea of what “targeted therapy” means in AML. Not all precision must be mutation-specific. Cell-surface antigens, differentiation state, and lineage-associated expression profiles can also define therapeutic vulnerabilities [9,13,14,36]. This point is especially relevant because AML often contains functionally important clones that do not map neatly onto a single dominant driver mutation [2,3,28]. Antibody–drug conjugates, bispecific approaches, and macrophage-engaging antibodies may therefore prove valuable even in genomically complex disease [9,28,36].

In current practice, gemtuzumab occupies a more selective role than FLT3 inhibitors, but its continued relevance is substantial [14,15,23,25]. In core binding factor AML and some intermediate-risk groups, it may enhance chemotherapy-based cure rates [14,15]. More broadly, it serves as proof that targeted intensification can be effective even when the target is present in a broad rather than genetically delimited population. This differs from the FLT3 model but complements it. AML precision medicine may ultimately require both mutation-defined and phenotype-defined targeting.

The future of antibody-based integration with intensive chemotherapy remains open. CD123-directed strategies, bispecific antibodies, and immune-engaging constructs are mostly earlier in development and have often been explored first in relapsed disease or low-intensity settings [9,28,36]. Yet the fit AML population may ultimately be one of the most attractive environments for these therapies if schedules can be engineered rationally. Intensive chemotherapy rapidly reduces burden; an antibody-based or immune-based agent could then be used to eliminate residual blasts or stem-like compartments that survive cytotoxic therapy. A useful parallel can be drawn from acute lymphoblastic leukemia, where intensive chemotherapy-based induction has increasingly been followed or complemented by biologically directed strategies, including tyrosine kinase inhibitors in molecularly defined subsets and bispecific T-cell engagers such as blinatumomab during consolidation. This model illustrates how cytoreduction, MRD clearance, and post-remission immune or targeted interventions may be distributed across distinct therapeutic phases rather than concentrated exclusively within induction. The challenge is timing. Immune-engaging agents may be difficult to deliver during profound aplasia, and antigen expression may shift under therapeutic pressure. Trial design will need to distinguish between agents best given concurrently with induction and those better reserved for consolidation or maintenance.

Thus, even though contemporary discussion often centers on kinase and metabolic inhibitors, antibody-based targeting remains integral to the broader history and future of AML combination therapy. Gemtuzumab showed that the fit AML setting can accommodate a targeted agent that changes relapse biology [14,15]. The next generation of antibody- and immune-based therapies will need to achieve the same while fitting more elegantly into the intensive treatment continuum.

## 5. IDH Inhibition, Venetoclax, and the Expansion of the Intensive Platform

If FLT3-mutated AML provided the first definitive model of genotype-directed intensive therapy, IDH-directed treatment and venetoclax-based regimens illustrate the next phase of platform expansion [5,6,19]. IDH1 and IDH2 mutations are biologically distinctive because they create neomorphic enzymatic activity leading to the accumulation of 2-hydroxyglutarate, altered epigenetic state, and impaired differentiation [5,36,37]. In lower-intensity settings, inhibitors of mutant IDH proteins have already demonstrated clinically meaningful activity, establishing differentiation-based therapy as a viable principle in AML [5,37]. The key question in fit patients is different: can IDH inhibition add something to intensive chemotherapy that chemotherapy itself does not already accomplish?

The answer remains unsettled, but the rationale is strong. Intensive chemotherapy is highly effective at reducing the proliferative bulk, while IDH inhibition may improve maturation and suppress the persistence of differentiation-blocked clones that survive treatment [5,36,37]. Importantly, IDH-mutated AML is not biologically uniform. Co-mutations in NPM1, DNMT3A, RUNX1, signaling genes, and splicing factors likely influence the value of adding an IDH inhibitor to chemotherapy [2,3,5,33,37]. This means that even if frontline IDH-directed intensive therapy proves beneficial overall, its greatest utility may emerge only once molecular context is incorporated more precisely into trial design. The field should avoid expecting a single global result to fully capture the value of these combinations [9,28,36].

Venetoclax raises even more provocative possibilities. In older or unfit patients, venetoclax-based low-intensity therapy has produced some of the most practice-changing advances in AML in years [20,33,38]. That success has naturally prompted efforts to move venetoclax earlier and into fitter populations receiving intensive regimens [6,21,34,35]. The biologic rationale is compelling. AML cells often display dependence on antiapoptotic proteins; venetoclax lowers the threshold for mitochondrial apoptosis and may therefore amplify the cytotoxic effect of chemotherapy [6,32,33,38]. In addition, some molecular subsets—including NPM1-mutated and IDH-mutated disease—appear especially apoptosis-sensitive, suggesting that venetoclax may not function merely as a generic intensifier but as a biologically tuned enhancer of depth of remission [6,32,33].

Early intensive venetoclax combinations have reported striking remission rates and high frequencies of MRD negativity [6,7,35]. These observations are exciting, but they must be interpreted with appropriate restraint. Deep remissions in early-phase studies do not automatically translate into superior long-term survival, especially if prolonged cytopenias, infectious complications, or treatment delays compromise delivery of consolidation or transplant [6,31,34,35,38]. Venetoclax also introduces practical complexities involving azole interactions, marrow recovery assessment, and schedule optimization [6,35,38]. It may be that the best use of venetoclax in fit AML is not continuous addition through every cycle, but carefully timed exposure that maximizes apoptosis during induction while minimizing prolonged aplasia.

One of the most interesting conceptual shifts produced by venetoclax is that it blurs the line between mutation-specific and state-specific targeting. BCL2 dependence is not a simple genomic lesion; it is a functional vulnerability that may vary by differentiation state, mitochondrial priming, and co-mutational architecture [32,33]. This opens the door to a more nuanced form of precision medicine in AML. The future may not rely only on whether a mutation is present, but on whether a leukemia displays a targetable survival state at a given time point in therapy.

Combining venetoclax with mutation-directed inhibitors and intensive chemotherapy is therefore an especially active area of interest [6,34,35]. FLT3-mutated AML is an obvious candidate because kinase inhibition and apoptosis priming may be synergistic [6,34]. IDH-mutated AML is another because differentiation effects and mitochondrial dependence may intersect in ways that make triplet therapy unusually potent [5,6,33,37]. Yet these regimens also crystallize the central dilemma of frontline AML innovation: every added biologic layer may improve leukemia control while simultaneously increasing regimen complexity. The correct metric of success is not only remission depth but whether the patient reaches the next planned treatment landmark-consolidation, transplant, or maintenance-on time and in acceptable condition.

For this reason, the intensive platform should be viewed as expandable but not infinitely so. Some targeted agents may be best integrated throughout induction and consolidation, as with FLT3 inhibitors. Others may prove most effective in a more selective manner, for example during induction only, as post-remission maintenance, or as MRD-directed intervention [6,19,22,26,29,31]. The ultimate contribution of IDH inhibitors and venetoclax to fit AML may therefore lie not in simply making standard chemotherapy stronger, but in making the entire frontline sequence more biologically coherent [9,28,36].

## 6. Menin Inhibitors and the Next Molecular Frontier

Menin inhibitors represent one of the most compelling new molecular strategies in AML, particularly in KMT2A-rearranged and NPM1-mutated disease, where leukemogenesis is sustained by aberrant HOXA/MEIS1 transcriptional programs [39,40]. In relapsed or refractory acute leukemia, revumenib has provided the clearest clinical proof of concept for pharmacologic menin inhibition, and ziftomenib has further supported menin as a tractable therapeutic target in genetically defined AML subsets [39,40]. The recent regulatory trajectory of revumenib has reinforced the relevance of this class: in November 2024, the FDA approved revumenib for relapsed or refractory acute leukemia with a KMT2A translocation, and in October 2025 its indication was expanded to relapsed or refractory AML with a susceptible NPM1 mutation [35,41].

**Figure 1:**
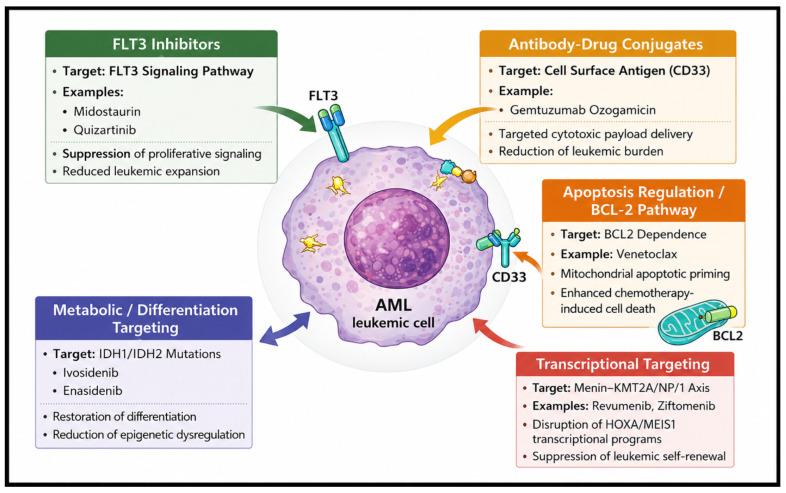
Major classes of targeted therapies currently integrated or under investigation in combination with intensive chemotherapy for acute myeloid leukemia. These agents target distinct biological vulnerabilities, including proliferative signaling (FLT3 inhibitors), cell surface antigens (gemtuzumab ozogamicin), metabolic and differentiation pathways (IDH inhibitors), apoptotic regulation (venetoclax), and transcriptional programs sustaining leukemic self-renewal (menin inhibitors).

For fit patients receiving intensive chemotherapy, however, the central question is not whether menin inhibition is biologically relevant, but how it should be incorporated into multi-phase frontline therapy. These agents are especially attractive because they target a transcriptional dependency rather than a conventional kinase pathway, potentially offering a means of suppressing leukemic self-renewal programs that persist despite cytoreduction [39,40]. That said, the frontline role of menin inhibitors remains investigational. Their eventual value may lie in molecularly selected induction combinations, in post-remission strategies aimed at deepening molecular clearance, or in MRD-directed approaches for patients with persistent NPM1-mutated or KMT2A-rearranged disease [7,40]. As with prior targeted platforms in AML, the decisive issue will be whether these agents improve durable disease control without compromising count recovery, transplant timing, or the deliverability of consolidation and maintenance (Table 1 and Table 2) (Figure 1).

## 7. Randomized Evidence, Endpoints, and What Really Changed Practice

A recurring problem in AML drug development is the temptation to overinterpret surrogate endpoints. Response rates matter, especially in aggressive disease, but the frontline fit population demands more stringent evidence than salvage settings [6,9,19,28]. A targeted agent that modestly increases complete remission without improving relapse-free or overall survival may not meaningfully change practice if it also increases toxicity or complicates transplantation [6,15,18]. The trials that have genuinely altered the standard of care did so because they affected the treatment course in a durable way, not merely the first marrow assessment [14,15,19,22].

This is why the timeline of targeted therapy with intensive chemotherapy is best understood as a progression in evidentiary rigor. The earliest antibody and kinase studies demonstrated feasibility and biologic activity [9,13,18]. Later randomized trials, especially those involving gemtuzumab ozogamicin and FLT3 inhibitors, began to show that selective biologic augmentation could change relapse risk or survival [14,15,19,22]. What changed practice was not simply the presence of a target, but the convergence of several elements: an adequately selected population, a clinically relevant endpoint, and a treatment design spanning induction and post-remission phases [9,19,22,28].

The fit AML setting also exposes the limitations of broad aggregate endpoints. Overall survival is essential, but it can be confounded by transplantation, crossover, relapse therapies, and evolving supportive care. Event-free survival is useful but may weigh induction failure, relapse, and death in ways that obscure the mechanism of benefit. MRD, cumulative incidence of relapse, and transplant-adjusted outcomes are increasingly important for interpreting why a targeted strategy succeeded or failed [7,8,31]. For instance, an agent may not dramatically increase remission rates yet still improve survival by reducing the quality-adjusted burden of relapse or by delivering more patients to transplantation in molecularly favorable states.

Another subtle but important issue is that frontline targeted therapy should be judged as a platform rather than as a fixed drug addition. Midostaurin changed practice not because it was the most potent FLT3 inhibitor ever developed, but because it was successfully embedded into a complete therapeutic sequence [19]. Similarly, the next wave of practice-changing trials may not necessarily identify the single strongest inhibitor in vitro; they may identify the regimen that best harmonizes induction efficacy, count recovery, transplant timing, maintenance feasibility, and management of molecular residual disease [6,26,29,31,35]. This is one reason why early enthusiasm based solely on potent single-agent activity in relapse can be misleading [27,39,40].

Practice-changing evidence in fit AML must also navigate the reality that standard chemotherapy is not static. Daunorubicin dosing, consolidation intensity, transplant availability, antifungal prophylaxis, and supportive care have all evolved [1,4,23,25,32]. Therefore, the benefit of a targeted agent is partly contextual. A combination that looks marginally beneficial against one historical backbone may perform differently when paired with contemporary supportive care and more precise molecular monitoring [7,8,25,31]. This is particularly relevant as MRD-guided transplant strategies become more common. A targeted therapy that increases MRD negativity may have amplified value in modern care pathways even if older trials were not designed to capture that effect explicitly [7,8,31].

Ultimately, the most useful interpretation of randomized evidence is pragmatic. Which targeted additions have crossed the threshold from promising to practice-defining? FLT3 inhibition clearly has [19,22,25,29]. Gemtuzumab ozogamicin, in selected risk groups, arguably has as well [14,15]. Which strategies remain compelling but not yet definitive? Intensive venetoclax combinations, frontline IDH inhibitor platforms, and menin-directed approaches belong in this category [5,6,34,35,39,40]. Which lesson should trialists carry forward? That the next generation of studies must not only test whether targeted therapy can be added, but determine how its benefit should be measured across the entire frontline continuum [7,8,31].

## 8. Resistance, Transplantation, and Maintenance Across the Treatment Continuum

No discussion of targeted therapy in fit AML is complete without addressing resistance, transplantation, and maintenance, because these determine whether early gains are converted into durable benefit [7,8,26,27,28]. AML relapse after combination therapy rarely reflects simple re-expression of the original target alone. More often, relapse is the product of clonal evolution, selection of pre-existing minor populations, acquisition of secondary mutations, adaptive rewiring of signaling networks, and persistence of stem-like compartments that are incompletely affected by both chemotherapy and targeted agents [2,3,8,27,28]. This is why frontline targeted therapy cannot be conceptualized as a one-step intervention. It must be understood as part of an evolving contest between treatment pressure and clonal adaptation.

In FLT3-mutated AML, the resistance story is particularly informative. Secondary kinase domain mutations, parallel pathway activation, and emergence of FLT3-independent clones all contribute to relapse [27,28,29]. This explains why potent FLT3 inhibition at diagnosis, while beneficial, is not by itself sufficient for many high-risk patients. Allogeneic transplantation remains a central consolidative strategy for a large subset of fit adults, especially those with biologically adverse disease or residual molecular uncertainty after induction [4,23,25,26]. The key modern question is no longer transplant versus targeted therapy, but how targeted therapy should optimize transplant outcomes. Can pre-transplant inhibitor exposure improve the depth of remission? Should post-transplant maintenance be routine in molecularly defined subgroups? How should MRD and mutation clearance guide duration? These are among the most clinically relevant unresolved issues in the field [7,8,26,29,31].

Maintenance has therefore moved from being a peripheral idea to a core component of frontline strategy. The traditional AML model viewed therapy as finite: induction, consolidation, transplant when indicated, then surveillance. Targeted therapy has destabilized that model. If relapse often originates from low-level residual clones, especially in molecularly defined disease, then maintenance or prolonged suppression becomes biologically plausible [7,8,26,31]. FLT3 inhibitors after transplant are the clearest example, but the principle could extend further [26,29]. Patients achieving remission with intensive chemotherapy plus a targeted agent may still harbor trace disease below morphologic detection. In that setting, continuation of targeted therapy may function not as overt treatment but as ecological control of residual malignant fitness. In FLT3-mutated AML, post-transplant maintenance is progressively being incorporated into the broader therapeutic strategy, reflecting a shift from induction-centered treatment toward a continuum of molecularly directed disease control. Rather than representing a separate intervention, maintenance therapy after allogeneic transplantation may be viewed as an extension of targeted treatment aimed at suppressing residual leukemic clones and reducing the risk of molecular relapse. The BMT CTN 1506/MORPHO trial, evaluating gilteritinib in this setting, provides important support for this concept, particularly in patients with evidence of peri-transplant MRD. These data underscore the potential role of FLT3 inhibitors not only in achieving remission before transplantation, but also in sustaining remission after transplant as part of an integrated, phase-adapted therapeutic approach.

The transplant interface also reveals why regimen tolerability matters as much as anti-leukemic potency. A combination that produces impressive marrow clearance but delays count recovery, increases invasive fungal infection, or causes cumulative organ toxicity may ultimately weaken the transplant pathway it seeks to improve [6,26,34,35,38]. This tension is especially important with venetoclax-augmented intensive regimens. Deep remission is desirable, but not at the cost of prolonged aplasia that compromises subsequent consolidation or delays donor procedures [6,34,35]. Therefore, the most clinically valuable combinations may be those with slightly less dramatic early response kinetics but more reliable pathway completion.

Resistance biology further argues for adaptive monitoring. Morphology alone is an inadequate lens through which to evaluate success in modern AML [7,8,31]. Serial mutation tracking, MRD assays, and perhaps eventually functional drug-sensitivity profiling may identify patients whose leukemia is escaping a targeted platform before frank hematologic relapse occurs. Such patients may benefit from early intensification, switch of inhibitor, or transplantation. In this model, frontline therapy becomes iterative rather than static.

The broader implication is that targeted therapy combined with intensive chemotherapy should no longer be evaluated only at the moment of induction. Its true value lies in how it shapes the entire continuum from diagnosis to post-remission surveillance. A regimen that produces a good remission but leaves no rational maintenance option may be less useful than one that establishes a coherent longitudinal strategy. The future of fit AML therapy will therefore depend not only on better drugs, but on better choreography of induction, transplant, maintenance, and molecular monitoring.

## 9. Future Directions

The future of targeted therapy with intensive chemotherapy in fit AML will likely be defined by three transitions: from single-target thinking to network-based combination design, from static baseline classification to dynamic molecular monitoring, and from regimen comparison to pathway optimization [9,28,43]. These transitions are already visible. Trials are increasingly asking not only whether a targeted drug improves induction results, but whether it changes the quality of remission, the biology of residual disease, the need for transplantation, and the feasibility of maintenance [7,8,26,31,34]. This is a more mature and more clinically relevant way to think about AML therapy.

The first transition is therapeutic. Single-agent augmentation has been necessary to establish proof of principle, but AML biology argues for rational combinations that address proliferation, apoptosis, differentiation arrest, and immune evasion together [9,28,34]. FLT3 inhibition plus chemotherapy was the first successful layer [19,22]. Venetoclax-based augmentation may become a second [6,34,35]. The next step could be triplet or sequence-adapted regimens in biologically selected subgroups, for example chemotherapy plus FLT3 inhibition plus venetoclax in highly proliferative FLT3-mutated disease, or chemotherapy plus an IDH inhibitor in patients whose leukemia shows persistent differentiation blockade despite cytoreduction [5,6,34,35,37]. Menin inhibitors may eventually enter the same framework in KMT2A-rearranged or NPM1-mutated AML, particularly if ongoing development confirms that they can deepen molecular responses without excessive additive toxicity [35,39]. The challenge will be to avoid simply making regimens more complex without making them more coherent. Rationality must remain biologically anchored and operationally feasible.

The second transition is diagnostic and monitoring-based. Baseline genomics remains essential, but it is no longer sufficient [2,3,25]. Two patients with the same nominal driver mutation may have very different clonal architectures, stemness programs, and probabilities of durable response [3,8,28]. As MRD assays improve and serial sequencing becomes more practical, treatment will increasingly be adapted based on early molecular kinetics rather than solely on morphology or static risk categories [7,8,31]. This may profoundly affect how targeted therapy is used. Some patients may need prolonged inhibitor exposure because molecular clearance is slow; others may safely de-escalate. Some may proceed directly to transplant because residual disease persists despite combination therapy; others may remain on chemotherapy-based pathways because targeted augmentation has converted a previously high-risk profile into a more favorable dynamic state.

The third transition is conceptual. Intensive chemotherapy has long been treated as the unquestioned backbone of fit AML treatment. That remains true today, but it may not remain true indefinitely [1,4,25,43]. The success of venetoclax in older adults, the maturation of mutation-directed therapy, and the emergence of menin-targeted and immune-based approaches all raise a provocative possibility: perhaps chemotherapy will evolve from being the core therapeutic identity of AML treatment to being one module among several [9,20,33,38,39]. The PARADIGM trial further challenges the conventional boundary between intensive and lower-intensity treatment in fit patients with newly diagnosed AML. By comparing azacitidine plus venetoclax with intensive chemotherapy, while allowing allogeneic transplantation in both arms, this study suggests that selected lower-intensity targeted strategies may achieve clinically meaningful remissions with reduced toxicity. In the near term, fit patients will almost certainly continue to receive intensive induction. However, over time, one can imagine more flexible pathways in which chemotherapy intensity is adapted to molecular risk, targetable vulnerability, and early response kinetics. Some patients may still need full-intensity cytotoxic debulking. Others may benefit more from targeted or immune-heavy combinations with less chemotherapy burden.

There is also a methodological imperative. Future frontline studies must resist the temptation to proliferate small, underpowered combinations that never answer decisive questions [9,28,43]. The field needs trials with clear biologic hypotheses, standardized supportive care, integrated MRD endpoints, and explicit plans for transplant and maintenance [7,8,31]. Without this, promising regimens risk becoming anecdotal rather than transformative. Cooperative group studies and thoughtfully designed platform trials will be crucial.

From an expert perspective, the main lesson of the last twenty years is that targeted therapy works in fit AML when it is integrated with discipline [9,14,15,19,22]. Success has depended on selecting the right disease state, the right target, the right exposure schedule, and the right longitudinal context. The field should therefore be ambitious but not indiscriminate. Not every active drug needs to be added to 7+3; some should replace components, some should follow induction, and some should be reserved for molecular persistence or relapse. Precision in AML will come not just from having more agents, but from knowing when not to use them [9,10,28,43].

The final question is therefore not whether targeted therapy belongs in frontline intensive AML; that has already been answered [14,15,19,22]. The real question is how far this logic can go. Can targeted combinations reduce the need for transplant in selected patients? Can MRD-guided therapy prevent overt relapse? Can biologically rational triplets improve survival without unacceptable toxicity? Can chemotherapy itself eventually be de-escalated in subsets where targeted and immune pressure are sufficient? These are not speculative curiosities; they are the practical agenda of the next decade [7,26,31,35].

Ultimately, targeted therapy combined with intensive chemotherapy should be viewed as a bridge between historical AML treatment and future precision oncology. The bridge has already been crossed in some subgroups and is under active construction in others. The task now is to ensure that future combinations are not merely more modern, but truly more curative.

## 10. Practical Implications for Current Frontline Care

For current clinical practice, the accumulated evidence suggests several practical principles [21,23,25,31]. First, molecular testing must be available rapidly enough to influence induction planning, because genotype-directed therapy is only useful if it reaches the patient at the relevant disease stage [5,19,22,25,35,37,39]. Second, targeted therapy should be planned across the entire treatment course rather than decided ad hoc at each cycle [19,22,26,29]. Third, the benefit of a targeted addition must be balanced against its effect on count recovery, infectious risk, antifungal compatibility, and transplant timing [6,26,34,35,38]. Fourth, MRD assessment should be incorporated whenever possible because it provides the clearest link between biologic depth of response and post-remission decision-making [7,8,31].

These principles also help explain why multidisciplinary AML care has become more important, not less, in the precision era [21,23,25]. Pharmacists, transplant physicians, molecular pathologists, and leukemia clinicians must coordinate around schedules, interactions, donor logistics, and molecular interpretation. The frontline targeted era is therefore not only about new drugs; it is about a new level of therapeutic orchestration. The regimens that succeed in practice will be those that are not merely biologically sophisticated, but operationally deliverable in real-world care.

## 11. Closing Perspective

Seen in this light, the past, present, and future of targeted therapy with intensive chemotherapy are not separate eras but connected stages of one therapeutic redesign. The past supplied cautionary lessons about pharmacology, schedule, and subgroup effect [13,18]. The present has delivered definitive successes, especially in FLT3-mutated AML, and credible signals in other biologic contexts [6,19]. The future will depend on whether the field can move from additive experimentation to genuinely adaptive precision strategy, now also including emerging transcriptional approaches such as menin inhibition in genetically selected AML subsets [7,31,35]. For fit AML patients, that shift could finally align initial remission quality with long-term cure probability in a way that conventional chemotherapy alone has rarely achieved [1,4,7,8].

## 12. Key Issues

- Intensive chemotherapy remains the standard backbone for medically fit AML, but targeted therapy has meaningfully modified frontline care in selected molecular subgroups [1,4,19,22,25].
- FLT3 inhibition provides the clearest proof that a targeted agent can improve survival when integrated with induction and consolidation chemotherapy [19,22,29].
- Gemtuzumab ozogamicin showed that phenotype-directed targeting can reduce relapse in selected patients when dose and schedule are optimized [14,15].
- IDH-directed therapy and venetoclax-based intensive regimens are promising, but their optimal roles likely depend on molecular context, sequencing, and post-remission strategy [5,6,33,34,35].
- Menin inhibitors are emerging as a highly promising strategy in KMT2A-rearranged and NPM1-mutated AML, but their definitive role in combination with intensive chemotherapy remains to be established [35,39].
- Measurable residual disease is increasingly central to judging the biologic depth of response and to guiding transplant and maintenance decisions [7,8,31].
- The future of fit AML therapy will likely depend on adaptive, longitudinal platforms rather than simple one-time drug additions [7,8,28,31,35].

## Figures and Tables

**Table 1:** Overview of targeted therapies combined with intensive chemotherapy in fit adults with acute myeloid leukemia (AML), including biologic rationale, clinical development stage, key efficacy signals, and major unresolved challenges.

Strategy/Agent Class	Biologic Target	Supporting Evidence	Clinical Setting in Fit AML	Clinical Relevance	Current Role in Clinical Practice	Limitations
Gemtuzumab ozogamicin (GO)	CD33-directed antibody-drug conjugate; phenotype-directed targeting of AML blasts	Sievers et al. [13]; ALFA-0701 [15]; Hills et al. meta-analysis [14]	Added to induction/consolidation chemotherapy in selected newly diagnosed fit adults	Reduced relapse risk and benefit in selected subgroups, especially favorable-risk and some intermediate-risk AML	Established in selected subgroups	Benefit is context-dependent; toxicity, especially hepatic toxicity/VOD risk, requires careful dose/schedule optimization
FLT3 inhibitors—midostaurin	Inhibition of constitutive FLT3 signaling in FLT3-mutated AML	RATIFY/Stone et al. [19]	Integrated across induction, consolidation, and continuation phases with intensive chemotherapy	Clear survival benefit in FLT3-mutated AML; proof of principle for genotype-directed frontline therapy	Practice-changing/standard of care in FLT3-mutated fit AML	Optimal integration with transplant, maintenance duration, and MRD-guided use still evolving
FLT3 inhibitors—quizartinib	Potent/selective FLT3 inhibition, particularly FLT3-ITD	Erba et al. [22]	Frontline combination with intensive chemotherapy in newly diagnosed FLT3-ITD AML	Reinforced frontline value of sustained FLT3 inhibition	Highly mature/practice-informing	Resistance mechanisms, clonal escape, sequencing with other FLT3 inhibitors, and post-transplant strategy remain unresolved
FLT3 inhibitors—gilteritinib	Potent FLT3 inhibitor with established activity in relapsed disease	Perl et al. [27]	Primarily relapsed/refractory AML; relevant as a candidate for earlier integration in fit AML platforms	Strong salvage efficacy; supports interest in earlier use	Promising, but frontline role not yet definitive in this review	Frontline benefit cannot be assumed from relapse efficacy; requires full-platform evaluation with chemotherapy
IDH inhibitors (ivosidenib, enasidenib)	Mutant IDH1/2 inhibition; reversal of 2-HG-driven differentiation block	DiNardo et al. [5]; Stein et al. [37]	Investigational addition to intensive chemotherapy in IDH1/2-mutated fit AML	Strong biologic rationale; promising activity extrapolated from non-intensive and relapsed settings	Promising but not practice-defining in fit AML	Uncertain incremental benefit over chemotherapy alone; effect likely depends on co-mutations, sequencing, and post-remission strategy
Venetoclax + intensive chemotherapy	BCL2 inhibition; apoptosis priming to deepen cytotoxic effect	Short et al. [7]; DiNardo et al. [35];	Investigational intensive combinations in newly diagnosed fit AML	High CR/MRD-negative rates in early studies	Promising but still investigational in fit AML	Cytopenias, infections, azole interactions, schedule optimization, transplant timing, and lack of mature long-term comparative survival data
Triplet strategies (e.g., chemotherapy + FLT3 inhibitor + venetoclax)	Simultaneous targeting of proliferation and apoptotic dependency	Discussed from [6,9,38]	Early-phase/frontier approach in biologically aggressive AML, especially FLT3-mutated disease	Potential for deeper remission and improved molecular clearance	Emerging/experimental	High regimen complexity, overlapping myelosuppression, feasibility, and whether deeper responses translate into better survival without impairing pathway completion
Menin inhibitors (revumenib, ziftomenib)	Menin inhibition in KMT2A-rearranged and NPM1-mutated AML; suppression of HOXA/MEIS1-driven leukemic programs	Issa et al. [39]; Wang et al. [40]; FDA approvals in R/R settings [42]	Currently investigational for frontline integration with intensive chemotherapy	Strong biologic and early clinical proof of concept in molecularly defined AML	Very promising, but early for frontline fit AML	Frontline role undefined; best use may be induction combination, post-remission deepening, or MRD-directed intervention
MRD-guided targeted strategy (cross-cutting concept)	Use of MRD/genomic monitoring to assess biologic depth of response and guide transplant/maintenance decisions	Short et al. [7]; Dillon et al. [8]	Across induction, consolidation, transplant, and maintenance in fit AML	Improves interpretation of response quality beyond morphology alone	Increasingly central, but not a drug class	Standardization, assay choice, timing, and how MRD should modify treatment remain active areas of development
Post-transplant/maintenance targeted therapy	Suppression of residual molecular disease after remission or allo-HSCT	Perl et al. [27]; Perl et al. [10]	Especially relevant in FLT3-mutated AML; concept potentially extendable to other molecular subsets	May prolong disease control after transplant and reduce relapse risk	Most mature in FLT3-mutated AML	Optimal duration, patient selection, MRD-guided discontinuation, and extension to non-FLT3 targets remain uncertain

**Table 2:** Key clinical studies discussed in this review, summarizing the development of targeted therapies and biologically informed treatment strategies in acute myeloid leukemia, with emphasis on their relevance to intensive frontline therapy in fit adults.

Study/Reference	Year	Study Design/Phase	Population/Setting	Investigational Strategy	Comparator/Backbone	Findings
Yates et al. [1]	1973	Pivotal chemotherapy study	Adult AML patients	Cytarabine + daunorubicin	None/historical backbone	Established the anthracycline-cytarabine backbone still used in intensive AML therapy
Sievers et al. [13]	2001	Clinical trial	Adult AML patients	Gemtuzumab ozogamicin	GO-based therapy	Early demonstration of CD33-targeted therapy activity in AML
Castaigne et al. (ALFA-0701) [15]	2012	Randomized clinical trial	Newly diagnosed Adult AML patients	GO + intensive chemotherapy	Standard chemotherapy	Fractionated GO improved outcomes in selected patients
Hills et al. [14]	2014	Meta-analysis	AML patients from randomized trials	GO + induction chemotherapy	Chemotherapy alone	GO reduced relapse risk in selected biologic subgroups
Röllig et al. [18]	2015	Randomized placebo-controlled clinical trial	Young newly diagnosed AML	Sorafenib + chemotherapy	Placebo + chemotherapy	Early attempt to integrate FLT3 inhibition in frontline therapy
Stone et al. [19] (RATIFY)	2017	Randomized phase III clinical trial	Newly diagnosed FLT3-mutated AML	Midostaurin + intensive chemotherapy	Placebo + chemotherapy	Landmark trial establishing FLT3 inhibition in frontline AML
Erba et al. [22]	2023	Randomized clinical trial	Newly diagnosed FLT3-ITD AML	Quizartinib + chemotherapy	Chemotherapy alone	Confirmed the benefit of potent FLT3 inhibition in frontline AML
Perl et al. [27]	2019	Randomized phase III clinical trial	Relapsed/refractory FLT3-mutated AML	Gilteritinib	Salvage chemotherapy	Demonstrated superiority of gilteritinib in relapse, supporting earlier use
DiNardo et al. [5]	2018	Clinical trial	Relapsed/refractory IDH1-mutated AML	Ivosidenib	Single-arm	Demonstrated clinical activity of IDH1 inhibition
Stein et al. [37]	2017	Clinical trial	Relapsed/refractory IDH2-mutated AML	Enasidenib	Single-arm	Established differentiation-based therapy targeting IDH2
DiNardo et al. [20]	2020	Randomized clinical trial	Previously untreated AML (mostly older/unfit)	Azacitidine + venetoclax	Azacitidine alone	Practice-changing regimen influencing venetoclax development in AML
Short et al. [7]	2020	Early-phase clinical study	Newly diagnosed AML	Venetoclax + intensive chemotherapy	Intensive chemotherapy	Reported high remission and MRD negativity rates
Short et al. [7]	2020	Clinical outcome study	AML patients with MRD assessment	MRD monitoring	Not a therapeutic comparison	Demonstrated strong prognostic value of MRD
Dillon et al. [8]	2023	Translational clinical study	Adult AML patients	DNA sequencing MRD detection	Conventional monitoring	Supported genomic MRD detection for residual disease
Issa et al. [39]	2023	Clinical trial	R/R leukemia with KMT2A-r or NPM1 mutation	Revumenib	Single-arm	Proof of concept for menin inhibition
Wang et al. [40] (KOMET-001)	2024	Phase I clinical trial	R/R Adult AML patients	Ziftomenib	Single-arm	Confirmed activity of menin inhibition in AML
Levis et al. [26]	2024	Clinical trial	Post-transplant FLT3-mutated AML	FLT3 inhibitor maintenance	Standard follow-up	Supports post-transplant targeted maintenance strategies
DiNardo et al. [35]	2024	Clinical trial	Newly diagnosed AML	Venetoclax + intensive chemotherapy	Intensive chemotherapy	Strengthens the rationale for venetoclax-based intensive combinations

## Data Availability

No new data were created or analyzed in this study.
